# Carriage of Multidrug-Resistant Bacteria in Healthy People: Recognition of Several Risk Groups

**DOI:** 10.3390/antibiotics10101163

**Published:** 2021-09-25

**Authors:** Christel Neut

**Affiliations:** U1286 INFINITE—Institute for Translational Research in Inflammation, Institut National de la Santé et de la Recherche Médicale (INSERM), Centre Hospitalier Régional Universitaire de Lille (CHU Lille), University of Lille, 59000 Lille, France; christel.neut@univ-lille.fr; Tel.: +33-3-20-96-49-36

**Keywords:** multidrug-resistant bacteria, healthy carriage, risk groups, prevention, eradication

## Abstract

The increase in multidrug-resistant (MDR) bacteria in hospitalized people and the hospital environment has been thoroughly documented. In contrast, little is known about their presence in the community. However, increasing evidence is showing a high level of carriage in people without infectious signs. Colonized people can later develop infections due to MDR bacteria and may be able to transmit them to susceptible people (the number of which is increasing worldwide), for example, people with comorbidities such as diabetes, cancer, or inflammatory diseases and those in extreme age groups. Risk factors for the acquisition of MDR bacteria are as follows: (1) residence or travel in countries with high levels of MDR bacteria; (2) occupational risks such as health workers or people with close contact with animals (farmers, veterinarians) who frequently use antibiotics; and (3) comorbidities. Eradication is rather difficult and, thus far, has not shown clear-cut results. Preventive measures will be important in the future with a reinforcement of hygienic measures not only in the hospital, but also in the community.

## 1. Introduction

The worldwide increase in multidrug-resistant (MDR) bacteria is a worrying phenomenon that is mainly associated with the lack of development of new antibacterial compounds. This increase is linked to the abundant use of antibiotics in humans as well as animals. This multidrug resistance concerns a large variety of microorganisms, both specific and opportunistic pathogens. Specific pathogens, such as *Mycobacterium tuberculosis* and *Neisseria gonorrhoeae*, do not develop in the human body without disease. In contrast, opportunistic pathogens can be found on different mucosal surfaces of the human body with the absence of any clinical signs. When these bacteria migrate to usually sterile sites, they can cause different types of infection. This type of evolution from healthy carriage to infection is typical for nosocomial infection.

The acronym ESKAPE includes six nosocomial pathogens that exhibit MDR and virulence: *Enterococcus faecium*, *Staphylococcus aureus*, *Klebsiella pneumoniae*, *Acinetobacter baumannii*, *Pseudomonas aeruginosa*, and *Enterobacter* spp. *Acinetobacter baumannii* and Pseudomonas aeruginosa rarely colonize the healthy human body. Staphylococcus aureus (including methicillin-resistant *Staphylococcus aureus* MRSA) frequently colonizes the human skin and the upper respiratory tract. *Enterococcus faecium* and enterobacterales (EB), such as Klebsiella pneumoniae and *Enterobacter* spp., and other EB, such as *Escherichia coli* (which is also increasingly becoming resistant to antibiotics), easily colonize the gastrointestinal tracts of healthy people.

While the carriage of MDR bacteria was limited to the hospital and to travelers coming back from countries with a high frequency of carriage at first, they are now appearing in the community with increasing frequency. The frequency and risk of transmission for MDR bacteria is not well known, as people with no clinical signs of infection are not screened or followed up by hospital staff.

A recent analysis (the first study of ESBL-producing *E. coli* in a healthy US population) revealed that half of healthy volunteers in a college community [1] harbored drug-resistant commensal Gram-negative bacteria in their guts. The risk of this was higher in females and after ATB intake.

One of the rare studies conducted on the carriage of intestinal MDR bacteria and changes over time was done in Paris and published in 2013 [2]. The French national health care system offers a medical examination (check-up) every five years. Three hundred and thirty-two people were checked for the carriage of extended-spectrum β-lactamase (ESBL)-producing *Escherichia coli* fecal isolate in 2006, and only 2 (=0.6%) were positive. Five years later, 345 people were examined and 21 positive carriers were identified, showing a tenfold increase. No links with travel, hospitalization, or antibiotic use were found. This convincing work (which only considered *E.coli*, while other EB, such as *Klebsiella* and *Enterobacter*, are more frequently MDR) shows a silent increase in MDR bacteria in a healthy population. Further study in this area will allow us to follow different risk groups more specifically.

Three risk groups are considered: The first is linked to the environment, underlining the risk of transmission. This concerns mother–infant transmission as well as transmission among children in daycare centers or among elderly living in long-term centers (both considered in chapter 4.3. as the health status of these individuals is also relevant). Geographical localization alone is also already an environmental risk factor, as travelers to countries with a high level of MDR bacteria are more likely to be exposed to transmission [3].

The second risk group is linked to occupational risk. This concerns health workers and all people working with animals (both farmers and the veterinary staff) [4].

Finally, the third group includes individuals whose health status influences the carriage of MDR bacteria. Underlying diseases, such as diabetes, increase the infection risk. This is probably linked to a higher carriage rate of potentially pathogenic bacteria [5]. Extreme age groups are also at a higher risk of transmission due to immune deficiency. In this paper, we also cover the risk of interhuman transmission. Additionally, underlying medical treatments, such as antibiotic (ATB) administration, can increase MDR colonization [3].

In practice, the risk factors of the three groups can be present at the same time in some populations, for example, diabetic travelers or travelers receiving ATBs as preventive or curative treatment for diarrhea.

As reports on the carriage of MDR bacteria in healthy people (as compared with MDR carriage in hospitalized patients with a high infection risk) are rare, the observations provided in this paper are often limited to a description, illustrating the lack of worldwide studies.

## 2. Environmental Risk

### 2.1. Geographical Localization

Differences in the frequency of isolation of MDR bacteria differ throughout the world and depend on sanitation conditions and antibiotic use. The best reports of carriers throughout the world are mainly based on ESBL-producing bacteria [2,6].

In particular, there is a high level of ESBL carriage in India, providing a basis for discussion on the risk of acquisition for travelers.

### 2.2. MDR Carriage in Travelers

International travel is becoming increasingly frequent and takes less time than it used to do before. The risk of MDR transmission is particularly linked to low-income countries with poor water quality and sanitation and uncontrolled use of antibiotics [5], inducing a higher level of antibioresistance. The influence of travel on the acquisition of MDR bacteria was first proposed based on clinical observations that patients with MDR infections were frequently travelers. Interesting trials confirmed this hypothesis; for example, a Swedish study [7] investigated the fecal carriage of ESBL-producing EB in travelers before and after their travel. After travel, 68 out of 226 people included in the study (who were not carrying ESBL-producing EB previously) tested positive for the presence of these bacteria, and it was considered that they had acquired these bacteria during travel. The main risk factors were age (people older than 65 years had a risk that was 7.38 times higher than that for people aged between 18 and 34 years old) and the country visited (travelers to Asia, especially the Indian subcontinent had a 6.64 times greater risk). Another recent study [8] followed 382 medical students who were completing internships abroad. Before their travel, the level of fecal carriage of ESBL-producing EB was 4.5%, and the mean value after travel was 29.3% (98.4% *E. coli*). The rate varied based on the country visited, from 10% in Tanzania to 40.8% in India, and 66.7% in Cambodia. The publication mentioned that probiotic administration had no effect on acquisition. This work illustrates a high acquisition rate in young, healthy people without major risk factors. Another study, which unfortunately had a small sample size (40 travelers), investigated the acquisition and persistence of ESBL-producing EB before and immediately after a trip to India and 3, 6, and 12 months later [9]. The rate of colonization before the trip was 10% and this increased to 76% after the trip, followed by a steady decrease (33% after 3 months, 26% after 6 months, and 18% after one year). Microbiota sequencing was done simultaneously, but did not reveal a statistically significant difference between ESBL-colonizers or non-colonizers. However, another study [10] revealed differences in the gut microbiota between carriers and non-carriers.

The same Swedish study group as above [11] evaluated the duration of colonization: 32% of post-travel carriers were still positive after 3 months, 25% after 6 months, 14% after 9 months, and 11% after 1 year. These are similar values to those presented above.

A recent systematic review [5] stated that in a study of 5253 travelers, 58.7% returning from South Asia were colonized by MDR EB. People with IBD and those using antibiotics, suffering from traveler’s diarrhea, or in contact with a health system overseas were found to have a higher risk of MDR acquisition. While the risk first concerned only ESBL-producing EB, colistin-resistant *E. coli* and carbapenemase-producing EB are now appearing more frequently [3], mainly in South America. Thirty-three percent of travelers remain positive after 3 months and 17% are positive after six months, emphasizing the high risk of transmission in the community throughout this time.

Diarrhea is the symptom most frequently associated with ESBL-carriage due to travel, and this is exacerbated by the intake of antibiotics. 

## 3. Occupational Risk

### 3.1. Hospitalized Patients and Health Workers

An important study in which 4376 patients were tested at the time of admission to hospital in six centers in Germany [12] revealed that 9.5% of patients were positive ESBL carriers, from which 79.1% of cases involved *E. coli*. The risk factors were previous antibiotic use, travel outside Europe, and a preceding stay in a long-term care facility (LTCF). This last factor may exacerbate the level of risk, because LTCF patients already have a higher risk of carriage than the general population.

An analysis of rectal swabs from 258 Spanish health workers revealed eight carriers of ESBL-producing EB [13].

One important meta-analysis [14] including data from 33,318 health workers taken from 127 studies showed that 4.1% of individuals had nasal carriage and 6.4% had hand carriage of MRSA, with identical levels among medical and nursing staff. Another study [15] followed 10 MRSA carriers in order to evaluate possible transmission to their family members. Transmission was confirmed in four cases.

The colonization of ESBL-producing EB was investigated in a multicenter study [16] involving five hospitals and 1001 health workers. Fecal carriage was identified in 3.5% of individuals studied, with all but one strain belonging to the species *E. coli.* The molecular analysis of patient and health worker strains did not confirm direct transmission of bacteria from patients to health workers, but rather, indicated the occurrence of healthy carriage, as it has been found in the community.

### 3.2. Activities Linked to Animals

Only poor descriptions of MDR carriage of veterinarians exist, suggesting that these are cases of colonization by identical MRSA clones [17]; however, systematic studies are needed.

Concerning farmers working with animals (frequently receiving ATBs) daily, one interesting work conducted in France compared pig farmers and non-farmers to investigate the nasal carriage of MRSA and the fecal carriage of enterococci and EB [4]. Nasal carriage of *Staphylococcus aureus* was found in 44.6% of farmers and only 24.1% of non-farmers (*p* < 0.01). Concerning antibiotic resistance, 72% of farmers were found to be carrying *Staphylococcus aureus* strains that were resistant to macrolides vs. 7.4% in non-farmers (*p* < 0.01). MRSA were found in 10% of farmers with no cases in non-farmers. The same clones were found in animals and humans, making a strong argument for direct transmission [18]. Analysis of the fecal carriage identified 69 out of 103 farmers tested as being positive carriers for streptomycin-resistant EB compared to 48 out of 100 non-farmers tested (*p* < 0.01). Tetracycline-resistant EB were found in 73 out of 103 farmers and 43 out of 100 non-farmers tested (*p* < 0.01). Another Dutch study [19] indicated that the MRSA clone ST398 is found frequently in calves and humans working in calf stables. The risk of colonization by this clone was found to be directly related to the number of hours worked by an individual.

The importance of ATB use was shown in a study conducted in Finland, a country with prudent ATB use in animals [20]. Among 320 veterinarians, only 3% carried ESBL-producing EB, and only one was colonized by MRSA. These frequencies are lower than has been reported for the global population in Finland.

## 4. Risk Linked to Health Status

Dysbiosis (microbiota different from what is found in people with a well-equilibrated microbiota) is a frequent disorder associated with an underlying immature or disturbed immune system, medical treatments, or diseases. People with dysbiosis are not always considered ill.

Temporary treatments (such as antibiotics) might also disturb the intestinal microbiota for short or long periods of time depending on the ATB administered. Another factor influencing dysbiosis without typical clinical signs is age (premature or young infants, the elderly). Immunosuppressive treatments can also alter the composition of the normal microbiota. The main diseases directly linked to dysbiosis are inflammatory bowel disease as well as ankylosing spondylitis and rheumatoid arthritis.

In all cases of dysbiosis, the penetration of exogenous bacteria is facilitated, which also leads to the introduction of MDR bacteria.

### 4.1. Underlying Disease

#### 4.1.1. Diabetes

Diabetic patients have an increased risk for all types of infection. The main life-threatening infections in diabetes are chronic wounds, which are responsible for the high rate of amputation in these individuals. These wounds are often infected with *Staphylococcus aureus*. A report on the cutaneous presence of *S. aureus* (susceptible or resistant to methicillin) indicated very high levels on the skin [21]. However, a detailed analysis of the different microbiota found in diabetic patients is still lacking.

#### 4.1.2. Inflammatory Bowel Disease

There is a lack of knowledge on MDR carriage in patients with ulcerative colitis or Crohn’s disease. However, these populations have risk factors associated with MDR carriage, such as frequent hospital stays, frequent surgeries, and long-lasting treatment with antibiotics or immunosuppressants. In a systematic review conducted by Furuya-Kanamori [5], travelers with inflammatory bowel disease were shown to have an increased risk of MDR carriage after travel.

#### 4.1.3. Cystic Fibrosis

**Figure 1:**
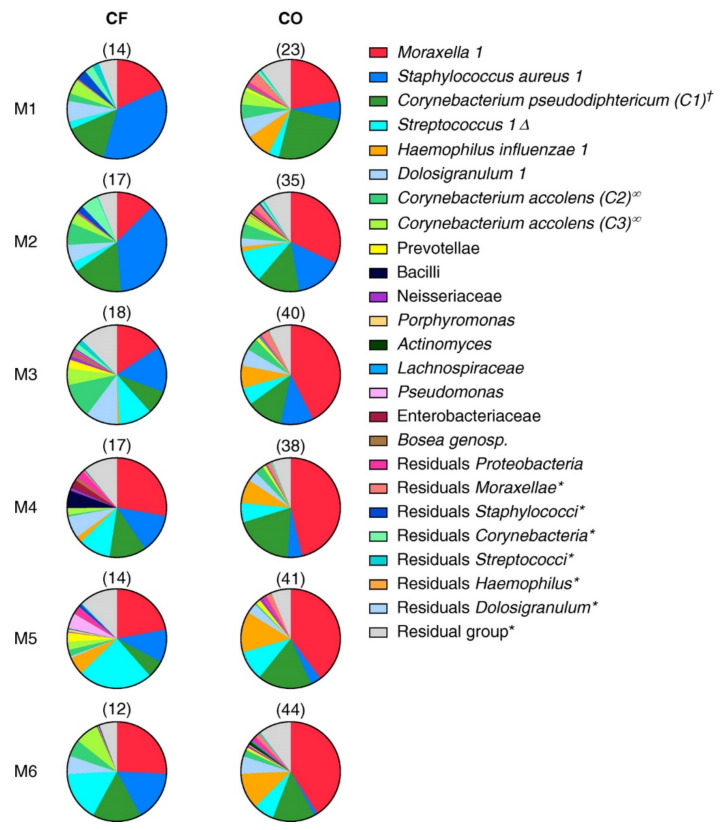
Overall development of the microbial community composition over time in infants with cystic fibrosis (CF) and control subjects (CO). The first and second columns of the pie charts show the development of the overall microbial community composition (top 35 operational taxonomic units [OTUs]) per month over the first 6 months (M) of life in infants with CF and healthy control subjects, respectively. The total number of samples included per month is depicted above the respective pie charts. For ease of visualization, the OTUs of the top 35 bacteria belonging to the same genus were merged. ^†^ 100% homology to *Corynebacterium pseudodiphtericum* and *Corynebacterium propinquum* after blasting. Δ *Streptococcus mitis* in CF and *Streptococcus pneumoniae* in control subjects determined by quantitative polymerase chain reaction, matrix-assisted laser desorption ionization time-of-flight mass spectrometry, and culture. ^∞^ 99% and 98% homology with *Corynebacterium accolens* after blasting. * All OTUs from the dataset belonging to this genus were included.

In children with cystic fibrosis, airway colonization is linked to both underlying disease and the regular consumption of antibiotics (as these children undergo significant antibiotic administration throughout their lives). The broad-spectrum antibiotics administered are potential inducers of MDR carriage, but differences in composition appear very early on in life (Figure 1). These are mainly linked to the early emergence of *Staphylococcus aureus* [22].

Differences in gut colonization also appear early on in life [23], leading to a high level of global diversity and a low level of bifidobacteria (normally predominant in children).

One recent work analyzed the resistome of feces from adult cystic fibrosis patients aged between 23 and 37 years (who had been subject to regular antibiotic consumption with various classes of antibiotics). The carriage of potentially transmissible resistance within the gut microbiomes of these patients was found to be significantly higher than that of healthy adults and could contribute to the emergence and dissemination of MDR pathogens [24].

#### 4.1.4. AIDS

In AIDS patients, the intestinal microbiota is characterized by decreased diversity, but an increased level of EB. Again, this is linked to a higher risk of MDR carriage (however, specific scientific results are scarce) [25].

#### 4.1.5. Gastroesophageal Reflux Disease (GERD)

The upper digestive tract is usually colonized by Gram-positive bacteria (usually also found in the mouth and the upper respiratory tract). Limited data on GERD patients with a global trend show a shift to Gram-negative anaerobes, but research on susceptibility to antibiotics is lacking [26,27].

### 4.2. Treatments

#### 4.2.1. Antibiotics

Antibiotics undoubtedly pose the greatest risk of dysbiosis, favoring the implantation of bacteria resistant to the administered antibiotics. Little data concerning the increase in MDR colonization exists, but for travelers, it has been found that the frequency of MDR carriage increases with antibiotic intake (for traveler’s diarrhea, for instance) [7,11]. As the modification of the microbiota is strongly influenced by the types of antibiotics taken, it is difficult to draw general conclusions.

#### 4.2.2. Gastric Acid Suppression

The administration of proton pump inhibitors (PPIs) was found to double the risk of acquiring MDR bacteria for patients [28]. This may be linked to reduced gastric acid secretion (increasing bacterial survival in the stomach), the subsequent modulation of the intestinal microbiota, or the presence of acid-resistant *E. coli*. PPI prescriptions should take this point into account.

#### 4.2.3. Immunosuppressive Treatments

Immunosuppressive treatments are used to treat a variety of diseases, including cancer and many inflammatory disorders, with increasing frequency. Specific results in this area are lacking, but it has been shown that the infection risk is increased.

### 4.3. Extreme Age Groups

#### 4.3.1. Premature Infants

Premature infants suffer from immaturity of most organs, but their development is also influenced by the hospital environment. The intestinal microbiota of these infants is enriched with facultative aerobes such as EB and *Enterococcus, Staphylococcus,* as well as clostridia (often with high virulence). Among EB, MDR bacteria are often [29] linked to frequent antibiotic use.

#### 4.3.2. Carriage of MDR Bacteria in Children

If MDR bacteria are able to colonize healthy adults (thought to have a balanced microbiota with reduced risk of colonization by exogenous bacteria), children with an imbalanced intestinal microbiota will have a higher risk of colonization. Indeed, there has been a worldwide increase in information on the high level of EBSL-producing *E. coli.* As *E.coli* (ESBL-producer or not) can always colonize the intestinal tract easily, it is not surprising that this species is of special concern regarding MDR carriage. Even in countries with reduced use of antibiotics, such as those in Africa, high levels of ESBL-producing *E. coli* are found. Up to 31% of infants under hospital admission in Niger carry these bacteria, but other underlying factors such as malnutrition can also be implicated [30].

The possibility of person-to-person transmission after prolonged contact was illustrated by an observation from a maternity hospital in Norway [31] where 26 pregnant women were found to be positive for fecal ESBL-producing bacteria. Fourteen were colonized at the time of childbirth, and of these, five had children who were colonized during the first days of life (transmission frequency 35.7%) and three of these were still colonized 2 months later. The PFGE profiles from the strains from the mothers and their infants were identical. Despite multiple analyses of breast milk from the colonized mothers, ESBL-producing bacteria were never found.

The carriage of ESBL-producing EB was investigated in 419 children in 25 daycare centers in Southeastern France in 2012. Twenty-eight strains were isolated (mean carriage rate 6.7%), from which 27 were identified as *E. coli* (and one strain of *Klebsiella pneumoniae)*. Scarce information on healthy children mentions that 1.7% of healthy preschool children in Peru and Bolivia in 2005, and 2.9% of healthy preschool children in Sweden in 2010 were found to be carriers [32]. In southern Taiwan, the prevalence of ESBL-producing *E. coli* was found to be 8.3% in 2014 [33].

#### 4.3.3. The Elderly

Elderly people have a higher infectious risk, due to their decreased immune function. The presence of MDR carriage in this population in the community is not well documented, but greater age is a constant risk factor in populations with high MDR carriage, such as travelers [34]. MDR carriage is thought be greater in residents of LTCFs, as antibiotic use is high among these populations. A study in Northern Ireland revealed the presence of ESBL-producing *E. coli* in the feces of 119 out of 294 patients studied (40.5%) [35]. Another research group [36] analyzed the influence of pet contact on MRSA carriage. Pet contact is often encouraged in nursing homes, but one argument against the presence of pets is the possibility of MDR bacteria transmission. MRSA were detected in 84 out of 229 (37%) residents living with pets and 99 out of 216 (46%) residents not living with pets. Thus, contact with healthy pets (not receiving antibiotics) is not a risk factor for MRSA carriage. Another study on MDR carriage in patients suffering from urinary tract infections (all taking ATBs) and their pets [37] revealed ESBL-producing *E. coli* in 35% of their pets (mainly dogs).

## 5. Preventive and Curative Measures

### 5.1. Curative Measures

#### 5.1.1. Selective Digestive Decontamination

Selective digestive decontamination aims to reduce the presence of aerobic MDR bacteria while maintaining the normal anaerobic part of the intestinal microbiota. It is indeed effective for decreasing the frequency of ventilator-associated pneumonia (one of the most important nosocomial infections linked to previous MDR carriage in the intestinal tract) [38]. However, a long-term effect of this type of treatment is an increase in MDR carriage due to the use of large-spectrum antibiotherapy [39].

#### 5.1.2. Inactivation of Antibiotics in the Intestinal Tract

As MDR carriage is the most often linked to the use of antibiotics, the inactivation of antibiotics in the intestinal tract seems a convenient way to limit this carriage [40]. Several attempts at developing a method to do this have been made, but it is difficult to find a unique compound that is able to inactivate all classes of antibiotics.

#### 5.1.3. Fecal Microbiota Transplantation

This type of treatment, which is usually not popular with patients, has become a convenient way to treat the recurrence of antibiotic-associated *Clostridium difficile* infection in the elderly. Some attempts have been made to apply this method for the case of MDR carriage, but the results have not been convincing. In a meta-analysis, the success rate was found to vary from 37.5% to 87.5% [41].

#### 5.1.4. Probiotic or Prebiotic Administration

The aim of probiotic treatments is usually to “improve” the intestinal microbiota through the administration of viable bacteria devoid of pathogenic potential. It is not well known whether these bacteria will compete with Gram-negative ESBL-producers. Further investigation is necessary to confirm whether treatment with probiotics can reduce MDR carriage [42]. Even though some probiotics are able to inhibit the growth of *Klebsiella pneumoniae* in vitro, there is no indication that the same actions occur in vivo (where pH shifts are not as easy as in in vitro conditions) [43].

Prebiotics (substrates for nonpathogenic bacteria such as lactobacilli or bifidobacteria) are being used in foods increasingly frequently to increase the levels of these bacteria in the intestinal lumen. However, no controlled trial has been conducted regarding their effect on MDR carriage. It is also not known if an increased level of these bacteria could preferentially compete with MDR bacteria rather than antibiotic-susceptible EB, which are present in healthy individuals at a medium level (while MDR bacteria may be present at low levels).

### 5.2. Preventive Measures

Only long-lasting measures are effective for the prevention of MDR carriage. Some preventive measures may be useful for avoiding or reducing the infection risk in patients (such as silver-coated endotracheal tubes, which reduce the introduction of harmful bacteria in the airways) [42].

Reducing the level of transmission of MDR bacteria from one patient to another or via medical staff is difficult to obtain at a long-lasting level. Alcohol-based hand gels may be useful for preventing transmission, but their effectiveness is reduced in people wearing rings or with fingernails more than 2 mm long [42].

Bacteriophages can selectively eliminate one type of previously identified bacteria, but they do not influence the remaining part of the intestinal microbiota [42].

#### 5.2.1. Education on Antibiotic Use: Antibiotic Stewardship

MDR emergence is the result of irrational use of antibiotics in humans, the veterinary field, and agriculture because of their easy and unregulated access, especially in developing countries [43]. Even in Europe, antibiotics are still overprescribed, and large-spectrum antibiotics are used too often. The medical staff (mainly general practitioners) is not sufficiently instructed about antibiotic prescriptions. Guidelines are often complex and not routinely used, while online training sessions seem to be more successful [44].

#### 5.2.2. Hygiene Improvement

Improved hygiene measures are needed not only in the hospital, but also in the community to reduce the potential risk of transmission to at-risk groups. This mainly concerns the risk of contact with fecal material from colonized persons. Educational measures should emphasize the importance of hand washing or the use of alcohol-based hand gels. The current coronavirus pandemic may have helped us adopt measures that will reduce transmission. 

## 6. Conclusions

The insidious carriage of MDR bacteria mainly in the intestinal tracts of healthy people in the community is an important threat to public health. Unfortunately, preventive and curative measures are rare, and the efficiency of developed methods needs to be confirmed. Only long-lasting measures (such as antibiotic stewardship or cleansing measures in the hospital) have positive impacts. We need more knowledge on the carriage of MDR bacteria in people with underlying diseases (who have an increased risk of MDR acquisition).

## Abbreviations

AIDS	Acquired immune deficiency syndrome
ATB	Antibiotic
EB	Enterobacterales
ESBL	Extended-spectrum beta-lactamase
GERD	Gastroesophageal reflux disease
LTCF	Long-term care facility
MDR	Multidrug-resistant
MRSA	Methicillin-resistant *Staphylococcus aureus*
PFGE	Pulsed-field gel electrophoresis
PPI	Proton pump inhibitor
